# Heterotopic Heart Transplantation as a Left Ventricular Biological Assistance: a New Two-Stage Method Proposal

**DOI:** 10.21470/1678-9741-2020-0506

**Published:** 2020

**Authors:** Fábio Antonio Gaiotto, Antonio Carlos de Almeida Barbosa Filho, Davi Freitas Tenório, Samuel Padovani Steffen, Fabio B. Jatene

**Affiliations:** 1 Cardiovascular Surgery Division, Instituto do Coração do Hospital das Clínicas da Faculdade de Medicina da Universidade de São Paulo (InCor-HCFMUSP), São Paulo, SP, Brazil.; 2 Centro Universitario CESMAC, Maceió, AL, Brazil.; 3 Hospital Israelita Albert Einstein - Pavilhão Vick e Joseph Safra, São Paulo, SP, Brazil.

**Keywords:** Heart-Assist Devices, Vena Cava, Superior, Ventricular Functional, Right, Quality of Life, Heart Transplantation, Ethics Committees, Atrophy

## Abstract

Since Barnard’s first heterotopic heart transplant in 1974, Copeland’s method has been the greatest contribution to heterotopic transplants but has the drawback of donor’s right ventricular atrophy. This new method proposes a modification in the anastomosis of the superior vena cava aiming to pre-serve donor’s right ventricular function by decompressing the pulmonary territory and reducing the pulmonary arterial pressure, as a biological ventricular assist device. Finally, a second intervention is proposed, where a “twist” is performed to place the donor’s heart in an orthotopic position after re-moval of the native heart. A pioneering research on this method received approval from the ethics committee of the Heart Institute of São Paulo. We believe that this method has the potential to im-prove quality of life in a selected group of patients.

**Table:** 

Abbreviations, acronyms & symbols	
HHT	= Heterotopic heart transplantation
IVC	= Inferior vena cava
LVAD	= Left ventricular assistance device
LV	= Left ventricle
OHT	= Orthotopic heart transplantation
PH	= Pulmonary hypertension
PVR	= Pulmonary vascular resistance
RV	= Right ventricle
SVC	= Superior vena cava

## INTRODUCTION

On December 3, 1967, Christiaan Barnard made history not only in cardiac surgery but also in medicine by doing the first orthotopic heart transplantation (OHT) in humans at Groote Schuur Hospital, Cape Town, South Africa. Barnard’s contributions were followed by the first heterotopic heart transplantation (HHT) with his own technique in 1974. The indications proposed by Barnard for HHT were, at that time: pulmonary hypertension (which is still a routine contraindication for OHT); systemic circulatory support for the native heart in primary graft failure and as circulatory support for the native heart in cases of severe rejection, the main cause of death in heart transplantation in the pre-cyclosporine era^[1,2]^.

Since these first three indications, two more were added: “small” donor hearts (in other words, a donor-recipient weight and height mismatch) and compromised donor hearts, also cited in the literature as “marginal” donor hearts, which means hearts that suffered a long ischemia time and are at high risk of complications, for example: hearts who needed high inotropic support, previous cardiac arrest or arrhythmia, abnormal wall motion visualized on echocardiogram and/or an electrocardiogram suggestive of ischemia^[1,2]^. It is worth mentioning that most donor hearts that present with those clinical scenarios are excluded for OHT donation.

**Fig. 1:**
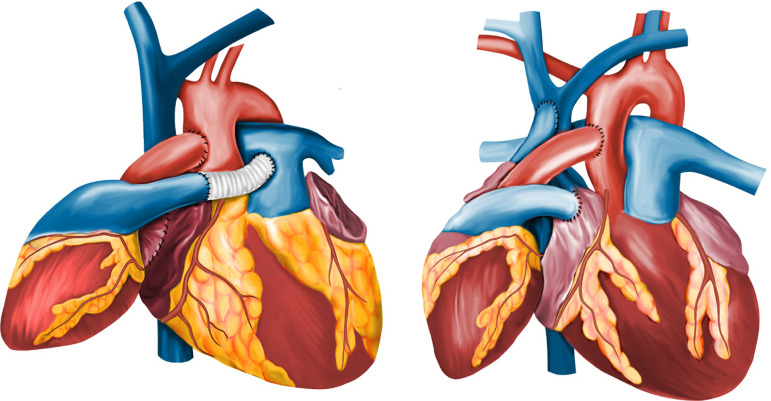
Final surgical aspect of heterotopic heart transplantation through Barnard’s technique (A) and through the original Copeland’s technique (B). Adapted from Copeland J, Copeland H^[1]^.

The use of HHT as left and right assistance or unilateral left assistance was supported by Barnard, although most patients were submitted to bilateral assistance exactly as the proposed technique that carries his name. The technique consists of na anastomosis between the left atrial chambers, followed by the anastomosis of the donor’s right atrium in the respective chamber and the superior vena cava of the recipient^[1,2]^. Then, na end-to-side anastomosis of the ascending aorta, finishing with the pulmonary trunk anastomosis to the respective recipient vessel with an interposed prosthetic tube (e.g., Dacron or heterologous pericardium), otherwise it would not be possible to connect them, at least not without tension (Figure 1A)^[2]^.

In Barnard’s technique for HHT, both circulations - right and left - are in parallel, which progressively turns the donor’s heart responsible for the entire blood flow and progressively reducing the activity of the recipient’s heart. This fact might lead to arrhythmias and other disastrous consequences, such as thrombus and embolus formation. Besides that, continuous dilation of the myocardium provides a favorable site for endocarditis. All these factors led to the discontinuation of Barnard’s technique.

As previously stated, OHT is contraindicated for patients with fixed pulmonary hypertension (PH) or elevated pulmonary vascular resistance (PVR) and other options for this select group of patients have emerged with time. One of them was the result of researches by Michael Ellis DeBakey and colleagues that culminated with the first successful left ventricular assistance device (LVAD) implantation in 1966^[3]^. These devices benefited a relevant pool of patients eligible for a bridge to transplantation, by providing a progressive reduction of PVR through the reverse remodeling of pulmonary artery branches, making these patients viable candidates for OHT.

The main obstacle for LVADs remains the high cost, restricting it to a limited number of patients and in developing countries, such as Brazil, this number is even lower. Nonetheless, heartlung transplantation, pioneered by Bruce Reitz and colleagues in 1981, is another option available, but isolated hearts are more readily available than heart-lung blocks, and fewer groups in the world have acceptable success rates with this procedure^[4]^.

Finally, a second surgical technique available for HHT was published by Jake and Hannah Copeland, and for this reason is called Copeland’s technique (Figure 1B). This technique provided left ventricular assistance, where both left ventricles were connected in a parallel circulation and, in addition, decompressed the right ventricle (RV) by connecting it to the circulation in series, which has been proven to work well in patients with good RV function, but with disappointing results in patients with biventricular failure^[5]^.

Analyzing the reported cases, Copeland’s HHT technique had unsatisfactory initial results, such as published in Copeland J and Copeland H^[1,2]^, suggesting as possible causes: poor adhesion to post-transplantation immunosuppressive treatment, mismatch between small donors and recipient’s heart, and marginal donor hearts. The latter is the most probable cause for the inferior results when compared to OHT^[6]^. Then, a new idea emerged.

The intention of this new two-stage approach is to assist the left ventricle (LV) by introducing a modification of Copeland's HHT technique, aiming to preserve the donor’s right ventricular function and decompress the pulmonary territory by reducing the pulmonary arterial pressure. After this pressure decrease, we propose the second stage by removing the native heart and “twisting” the donor’s heart to the orthotropic position, releasing the patient from the inherent risks of the dependence on good function of two hearts.

**Fig. 2:**
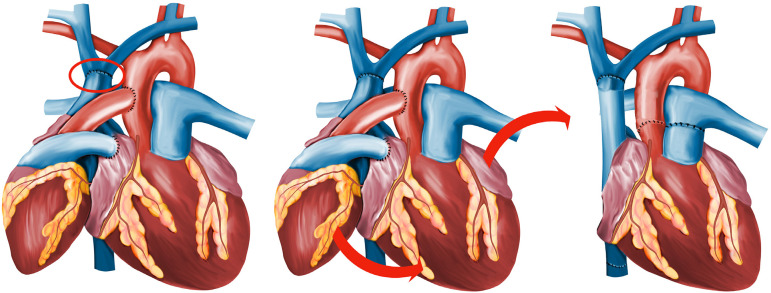
Final surgical aspect of heterotopic heart transplantation through modified Copeland’s technique with SVC end-to-end anastomosis providing an in series circulation on the right side and in parallel circula-tion on the left side (A), donor heart repositioning during autotransplantation (B) finally at (C) the donor heart after the “twist” and native heart removal.

The first stage starts with the anastomosis between left atria and ascending aorta, and then the anastomosis from donor's pulmonary trunk and recipient’s right atrium. Considering that the RV is a flow-dependent chamber, its function would be preserved, as it will receive all the flow from the total superior vena cava (SVC) venous return, contrasting the RV atrophy associated with the reduced flow from the side anastomosis of the original Copeland's technique, and this benefit would be attained by the direct end-to-end anastomosis of the donor SVC to the recipient SVC close to the brachiocephalic veins (Figure 2A), while closing the donor inferior vena cava (IVC) and the recipient SVC near to the right atrium taking care to avoid lesion of the sinoatrial node.

These modifications would allow the preservation of the RV function, while decreasing the PVR through the parallel anastomosis of the LV, creating the ideal environment for the previously cited “twist” of the donor heart in the second stage (Figure 2B and 2C). The pioneering research on this new method, proposed by Fabio Antonio Gaiotto^[7]^, from the Heart Institute of Sao Paulo, was approved by the institution's ethics committee (Approval Number: 4.709.109).

It is also interesting to note that the measured central venous pressure will have two distinct values: if measured in the SVC venous return territory (*e.g*., jugular vein catheter) it will reflect the donor's heart right circulation pressures; alternatively, if measured in the IVC venous return territory (*e.g*., femoral vein catheter), it will reflect the recipient's heart right circulation pressures. Another important remark is that the ideal pathway for the donor's heart biopsy will be the SVC, as it will be directly connected to the donor's right ventricle.

In conclusion, we believe that this new method has the potential to become a viable alternative for surgery in a selected group of patients, reducing the poor long-term outcomes and substantially improving their quality of life in this patient pool, who are mostly in palliative care in developing countries like Brazil.

**Table:** 

**Authors' roles & responsibilities**	
FAG	Substantial contributions to the conception or design of the work; or the acquisition, analysis, or interpre-tation of data for the work; drafting the work or revising it critically for important intellectual content; final ap-proval of the version to be published
ACABF	Substantial contributions to the conception or design of the work; or the acquisition, analysis, or in-terpretation of data for the work; drafting the work or revising it critically for important intellectual content; final approval of the version to be published
DFT	Substantial contributions to the conception or design of the work; or the acquisition, analysis, or interpre-tation of data for the work; drafting the work or revising it critically for important intellectual content; final ap-proval of the version to be published
SPS	Substantial contributions to the conception or design of the work; or the acquisition, analysis, or interpre-tation of data for the work; drafting the work or revising it critically for important intellectual content; final ap-proval of the version to be published
FBJ	Substantial contributions to the conception or design of the work; or the acquisition, analysis, or interpre-tation of data for the work; drafting the work or revising it critically for important intellectual content; final ap-proval of the version to be published
